# The Physical Location of Stripe Rust Resistance Genes on Chromosome 6 of Rye (*Secale cereale* L.) AR106BONE

**DOI:** 10.3389/fpls.2022.928014

**Published:** 2022-06-29

**Authors:** Yanling Duan, Jie Luo, Zujun Yang, Guangrong Li, Zongxiang Tang, Shulan Fu

**Affiliations:** ^1^College of Agronomy, Sichuan Agricultural University, Chengdu, China; ^2^Provincial Key Laboratory for Plant Genetics and Breeding, Sichuan Agricultural University, Chengdu, China; ^3^Center for Informational Biology, University of Electronic Science and Technology of China, Chengdu, China

**Keywords:** wheat, rye, stripe rust, 6R chromosome, small-segment translocation

## Abstract

It was reported that the chromosome 6R of rye (*Secale cereale* L.) carries stripe rust resistance gene *Yr83*, and the region with the candidate resistance gene(s) still needs to be narrowed down. This study confirmed that the chromosome 6RL^Ar^ derived from rye AR106BONE contains stripe rust resistance gene(s). A wheat-rye T6BS.6RL^Ar^ translocation chromosome, a wheat-rye small-segment translocation T6RL^Ar^-6AS.6AL, and three kinds of deleted T6BS.6RL^Ar^ translocations, T6BS.6RL^Ar^-1, T6BS.6RL^Ar^-2, and T6BS.6RL^Ar^-3, were identified. Translocations T6BS.6RL^Ar^, T6BS.6RL^Ar^-2, and T6RL^Ar^-6AS.6AL were highly resistant to stripe rust and T6BS.6RL^Ar^-1 and T6BS.6RL^Ar^-3 were highly susceptible. The molecular markers specific to 6RL determined that the three regions of the 6RL^Ar^ arm from 732,999,830 bp to the telomere, from 735,010,030 to 848,010,414 bp, and from 848,011,262 bp to the telomere were deleted from T6BS.6RL^Ar^-1, T6BS.6RL^Ar^-2, and T6BS.6RL^Ar^-3, respectively. T6BS.6RL^Ar^-2 and T6RL^Ar^-6AS.6AL contained the segment that was deleted in T6BS.6RL^Ar^-3. Therefore, it can be concluded that about 37 Mb segment from 848,011,262 bp to the telomere carried stripe rust resistance gene(s), and it was smaller than that with the *Yr83* gene. Gene annotation indicated that about 37 Mb region contains 43 potential resistance genes, and 42 of them are nucleotide-binding site and leucine-rich repeat (NBS-LRR)-like resistance protein genes. The results in this study narrowed down the size of the region with candidate stripe rust resistance gene(s) on the 6RL arm, and the T6RL^Ar^-6AS.6AL is a promising small-segment translocation for improvement of wheat cultivars.

## Introduction

Wheat stripe rust is caused by *Puccinia striiformis* f. sp. *tritici* (Pst) and is one of the most serious diseases in wheat. Developing wheat cultivars with resistance to stripe rust is the most practical way to control this disease. More than 80 *Yr* (yellow rust resistance) genes were officially named; however, most of them lost their resistance because of variations in the prevalence of virulent pathotypes (Ren et al., 2022). The resistance to stripe rust of 103 wheat lines was tested using pathogenic races CYR32, CYR33, and CYR34, which are currently prevalent in China, and only *Yr5, Yr15*, and *Yr45* exhibited all-stage resistance, and only *Yr41, Yr47*, and *Yr50* were adult plant resistant (Hu et al., 2022). The replacement of historically clonal *Pst* races by new ones occurred continually (Jamil et al., 2020; Bouvet et al., 2022). Therefore, it is important to explore new resistance genes to stripe rust and enrich the resource pool for wheat resistance breeding.

Wheat-related species contain abundant resistance genes. For example, rye (*Secale cereale* L.) is an important gene source for wheat disease resistance breeding (Spetsov and Daskalova, 2022). Stripe rust gene *Yr 9* located on the 1RS arm was successfully used in commercial wheat cultivars. Additionally, 2R, 4R, 5R, 6R, and 7R chromosomes also carry stripe rust-resistant genes (Lei et al., 2011; Li et al., 2016a, 2020a,b; Schneider et al., 2016; An et al., 2019; Xi et al., 2019; Johansson et al., 2020; Ren et al., 2020). Four of these reports indicated that rye chromosome 6R carried stripe rust resistance genes, and they were derived from different rye sources (Schneider et al., 2016; Johansson et al., 2020; Li et al., 2020a,b). A new stripe rust resistance gene on chromosome 6R was named *Yr83*, and it was located in a bin FL0.73-1.00 of 6RL (Li et al., 2020b). To clone the stripe rust resistance gene(s) on 6RL, it is necessary to narrow the segment with resistant gene(s). In this study, a wheat-rye 6R^Ar^ monosomic addition line (MA6R^Ar^) with resistance to stripe rust, a 6RS^Ar^ monotelosomic addition line (MTA6RS^Ar^), a 6RL^Ar^ monotelosomic addition line (MTA6RL^Ar^), a T6BS.6RL^Ar^ translocation line, and three kinds of deleted T6BS.6RL^Ar^ translocation lines were used to locate the stripe rust resistance gene(s) on a smaller segment of 6RL^Ar^ arm, and some resistance protein genes in this segment were found.

## Materials and Methods

### Plant Materials

Octoploid triticale lines Mianyang 11/AR106BONE-4 (MAR4) and Mianyang 11/Kustro (MK) were derived from common wheat (*Triticum aestivum* L.) Mianyang 11 (MY11) × *Secale cereale* L. AR106BONE and MY11 × *Secale cereale* L. Kustro, respectively. Crossing between MAR4 and a wheat line J1025 (*T. aestivum* L.) and between MK and MY11 was carried out. From the progenies of MAR4 × J1025 and MK × MY11, wheat-rye 6R monosomic addition lines MA6R^Ar^ and MA6R^Ku^ were identified, respectively. These monosomic addition lines were used to investigate the stripe rust resistance of the two kinds of 6R chromosomes. MTA6RS^Ar^ and MTA6RL^Ar^ were identified from the selfed progeny of MA6R^Ar^. The seeds of MA6R^Ar^ were irradiated with ^60^Co-γ rays at a dosage of 200 Gy at Biotechnology and Nuclear Technology Research Institute, Sichuan Academy of Agricultural Sciences, China. From the progeny of irradiated MA6R^Ar^, a wheat-rye T6BS.6RL^Ar^ translocation line was obtained, and the seeds derived from this translocation line were also irradiated with ^60^Co-γ rays and some deleted T6BS.6RL^Ar^ translocation chromosomes were detected. The rye Kustro and AR106BONE and the common wheat MY11 and J1025 were kept in the seed store in our laboratory.

### Cytological Analysis

The root-tip metaphase chromosomes of the materials used in this study were analyzed using non-denaturing fluorescence *in situ* hybridization (ND-FISH) technology. Oligo-pSc200, Oligo-pSc250 (Vershinin et al., 1995; Fu et al., 2015), Oligo-Ku (Xiao et al., 2017), Oligo-pSc119.2-1 (McIntyre et al., 1990; Tang et al., 2014), and Oligo-pTa535-1 (Komuro et al., 2013; Tang et al., 2014) were used as probes. Probes Oligo-pSc200, Oligo-pSc250, and Oligo-Ku were used to distinguish rye chromosomes from wheat (Fu et al., 2015; Xiao et al., 2017). Probes Oligo-pSc119.2-1 and Oligo-pTa535-1 were used to identify individual wheat chromosomes (Tang et al., 2014). Additionally, the different cytological structure of rye chromosomes was displayed by the combination of probes Oligo-pSc200, Oligo-pSc250, and Oligo-pSc119.2-1 (Fu et al., 2015). These oligonucleotide (oligo) probes were 5′ end-labeled with 6-carboxyfluorescein (6-FAM) or 6-carboxytetramethylrhodamine (TAMRA). The root-tip metaphase chromosomes were prepared according to the methods described by Han et al. (2006). The ND-FISH analysis was carried out following the methods described by Fu et al. (2015) and Xiao et al. (2017). Additionally, the genomic DNA of rye AR106BONE was used as a probe for the genomic *in situ* hybridization (GISH) analysis of the T6RL^Ar^-6AS.6AL small-segment translocation. The GISH analysis was carried out according to the methods described by Fu et al. (2015).

### Development of 6RL-Specific Markers

Primers were designed according to the 6RL sequence of rye Lo7 (Rabanus-Wallace et al., 2021) using Primer 3 software (version 4.0), and the optimal melting temperature and size values were set to 60°C and to 20 bases, respectively. In total, 423 pairs of primers were designed. Additionally, 124 developed 6RL^Ku^-specific length amplified fragment sequencing (SLAF-seq) markers (Li et al., 2016b) were also used. The PCR amplification and agarose gel electrophoresis were performed following the procedure described by Li et al. (2016b). Chinese Spring (CS), MY11, T6BS.6RL^Ar^ translocation line, and rye AR106BONE were used to test the 6RL^Ar^ specificity of the 423 newly designed primer pairs and the 124 6RL^Ku^-specific SLAF-seq markers. All the 6RL^Ar^-specific markers were physically mapped to specific regions of the 6RL^Ar^ arm using deleted T6BS.6RL^Ar^ translocation lines. The sequences of the 124 SLAF-seq primer pairs were used for nucleotide Basic Local Alignment Search Tool (BLAST) searches against the 6R sequence of rye Lo7 (Rabanus-Wallace et al., 2021) using a BLAST tool in the Triticeae Multi-omics Center (http://202.194.139.32/), and the positions of these markers on 6RL arm were determined according to the BLAST results.

### Cloning Partial Sequences of the Candidate Resistance Genes From 6RL^Ar^

To confirm that the candidate resistance genes on the 6RL arm of Lo7 are similar to that of 6RL^Ar^, the sequences of two candidate resistance genes *SECCE6Rv1G0449960.1* and *SECCE6Rv1G0453070.1* of Lo7 were randomly selected for designing primer pairs. Primer pairs of P60 (5′ TGGGG AATAG CTGGC ATTGG3′, 5′ TCGGT AGGGT AGACG GTGAG3′) and P70 (5′ AATGG GAGGA CTCTT GCGTG3′, 5′ CTGGG AATGA ACCGA CAGCT3′) were used to amplify the partial sequences of the two genes from 6RL^Ar^ arm. The PCR reactions and product separation were also performed according to the methods described by Li et al. (2016b), and the annealing temperature of the primer pairs was 60°C. The target products amplified by the two primer pairs, P60 and P70, were cloned and then sequenced by the Tsingke Biotechnology Co., Ltd. (Chengdu, China). Sequence alignment was performed using the software DNAMAN (Ver. 4.0, Lynnon Corp., Quebec, QC, Canada).

### Stripe Rust Response Test

The response of parental wheat J1025, MY11, lines MA6R^Ku^, MA6R^Ar^, MTA6RS^Ar^, MTA6RL^Ar^, T6BS.6RL^Ar^, and the deleted T6BS.6RL^Ar^ to stripe rust was evaluated. The mixed stripe rust prevalent isolates CYR32, CYR33, and CYR34 were used to inoculate seedlings in field according to the method described by Xi et al. (2019). A 0–9 numerical scale of infection types (IT) was scored according to the standard described by Wan et al. (2017) at the adult stage. The disease resistance of J1025, MY11, the lines MA6R^Ku^, MA6R^Ar^, MTA6RS^Ar^, MTA6RL^Ar^, and T6BS.6RL^Ar^ was evaluated in 2018–2019 in Qionglai, Sichuan Province, China and in 2019–2020, 2020–2021, and 2021–2022 in Wenjiang and Dayi, Sichuan Province, China. The disease resistance of the deleted T6BS.6RL^Ar^ and the T6RL^Ar^-6AS.6AL translocation was evaluated in 2020–2021 and 2021–2022 in Wenjiang and Dayi. When the segment of 6RL^Ar^ with stripe rust resistance was identified, the candidate resistance genes were found according to the gene annotation of rye Lo7 (http://202.194.139.32/jbrowse.html) (Rabanus-Wallace et al., 2021).

## Results

### Identification of Wheat-Rye 6R Addition and Translocation Lines

**Figure 1:**
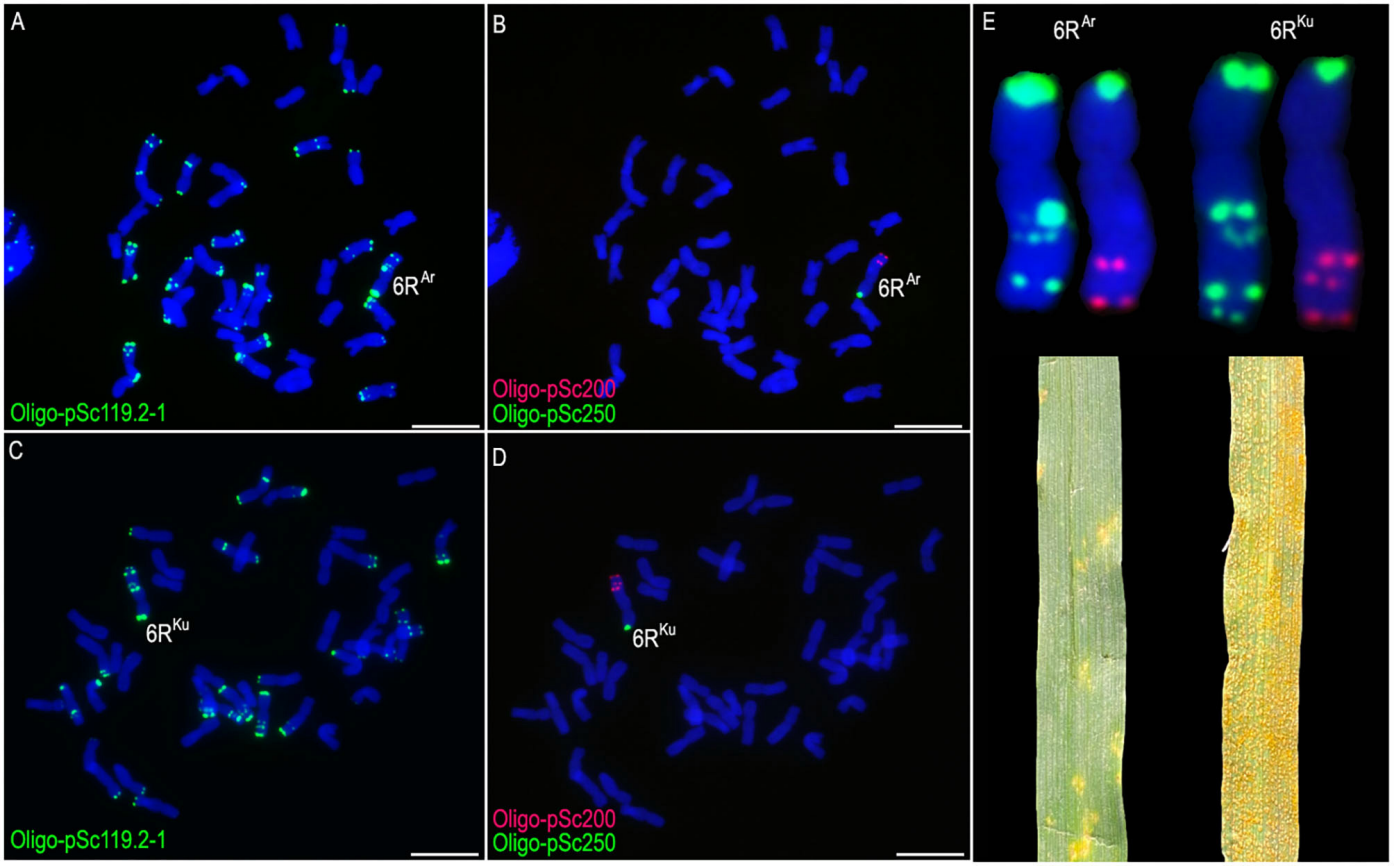
The non-denaturing fluorescence *in situ* hybridization (ND-FISH) analysis of lines MA6R^Ar^ and MA6R^Ku^ using probes Oligo-pSc119.2-1 (green), Oligo-pSc200 (red), Oligo-pSc250 (green), and their stripe rust resistance test. **(A,B)** A root-tip metaphase cell of line MA6R^Ar^. **(C,D)** A root-tip metaphase cell of line MA6R^Ku^. **(E)** Cut-and-paste 6R^Ar^ and 6R^Ku^ chromosomes. 6R^Ar^ was highly resistant to stripe rust and 6R^Ku^ was susceptible. Scale bar: 10 μm.

The ND-FISH based on oligo probes was used to analyze the progeny of wheat × rye. Wheat-rye 6R monosomic addition lines MA6R^Ar^ and MA6R^Ku^ were identified (Figures 1A–D). Both the short arms of the 6R^Ar^ and 6R^Ku^ chromosomes (6RS^Ar^ and 6RS^Ku^) contained signals of Oligo-pSc119.2-1 and Oligo-pSc250, and no signals of Oligo-pSc250 were observed on their long arms (6RL^Ar^ and 6RL^Ku^) (Figure 1E). The probe Oligo-pSc119.2-1 produced three and four signal bands on 6RL^Ar^ and 6RL^Ku^, respectively (Figure 1E). Two and three signal bands of probe Oligo-pSc200 were observed on 6RL^Ar^ and 6RL^Ku^ arms, respectively (Figure 1E). The results indicated that the structure of the long arms of the 6R^Ar^ and 6R^Ku^ chromosomes is different. Line MA6R^Ar^ was highly resistant to stripe rust (IT = 1), whereas line MA6R^Ku^ was susceptible (IT = 9; Figure 1E).

**Figure 2:**
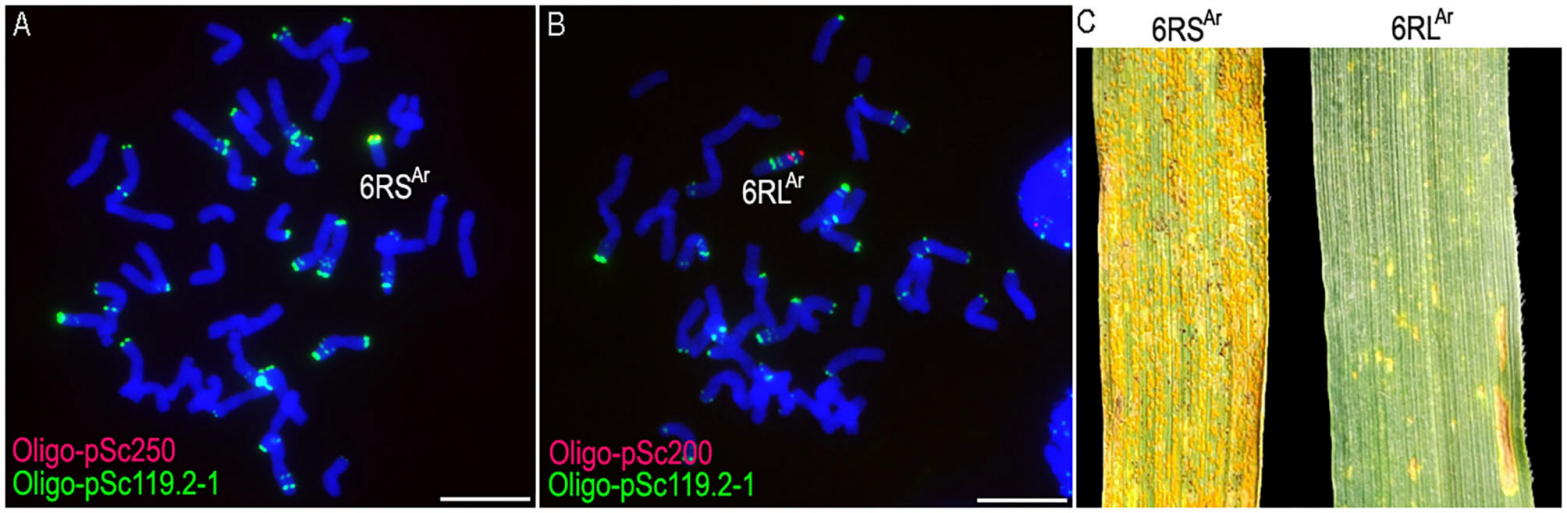
The non-denaturing fluorescence *in situ* hybridization (ND-FISH) analysis of lines MTA6RS^Ar^ and MTA6RL^Ar^ using probes Oligo-pSc119.2-1 (green), Oligo-pSc200 (red), Oligo-pSc250 (red), and their stripe rust resistance test. **(A)** A root-tip metaphase cell of line MTA6RS^Ar^. **(B)** A root-tip metaphase cell of line MTA6RL^Ar^. **(C)** Line MTA6RS^Ar^ was susceptible to stripe rust and line MTA6RL^Ar^ was highly resistant. Scale bar: 10 μm.

**Figure 3:**
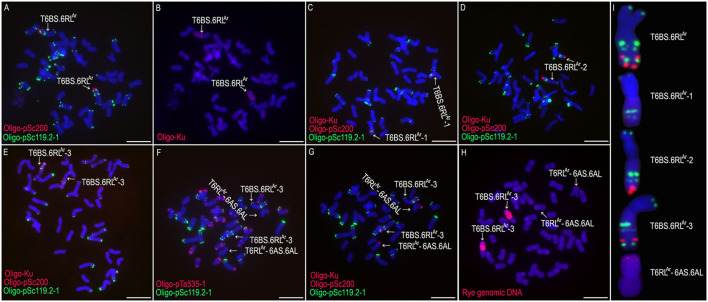
The non-denaturing fluorescence *in situ* hybridization (ND-FISH) and genomic *in situ* hybridization (GISH) analyses of T6BS.6RL^Ar^ translocations and its deleted T6BS.6RL^Ar^ using probes Oligo-pSc119.2-1 (green), Oligo-pTa535-1 (red), Oligo-pSc200 (red), Oligo-Ku (red) and rye genomic DNA (red). **(A,B)** A root-tip metaphase cell of line 19T177-21. **(C)** A root-tip metaphase cell of line 21F1. **(D)** A root-tip metaphase cell of line 21F3. **(E)** A root-tip metaphase cell of line 21F7. **(F,G)** A root-tip metaphase cell of line 21F11. **(H)** GISH analysis of root-tip metaphase cell of line 21F11. **(I)** Cut-and-paste chromosomes of T6BS.6RL^Ar^ and deleted T6BS.6RL^Ar^. Scale bar: 10 μm.

From the selfed progeny of line MA6R^Ar^, the 6RS^Ar^ and the 6RL^Ar^ monotelosomic addition lines, MTA6RS^Ar^ and MTA6RL^Ar^, were identified (Figures 2A,B). Line MTA6RS^Ar^ was susceptible to stripe rust (IT = 9), and line MTA6RL^Ar^ displayed high resistance (IT = 1; Figure 2C). In total, 200 seeds of MA6R^Ar^ were irradiated. A plant 18T231-46 containing a wheat-rye T6BS.6RL^Ar^ translocation chromosome was identified from 1,182 M1 seeds, and a line 19T177-21 containing a pair of T6BS.6RL^Ar^ translocations was identified from 30 seeds of the selfed progeny of 18T231-46 (Figures 3A,B). Additionally, 104 seeds of the selfed progeny of 18T231-46 were also irradiated. Four lines, 21F1, 21F3, 21F7, and 21F11, were identified from the 1,090 seeds (M2) of the progeny of irradiated 18T231-46. In these lines, three kinds of deleted T6BS.6RL^Ar^ translocation chromosomes and a small-segment translocation T6RL^Ar^-6AS.6AL were found (Figures 3C–I). Line 21F1 contained two deleted translocation chromosomes T6BS.6RL^Ar^-1, on which the Oligo-pSc200 signals and the distal Oligo-pSc119.2-1 signals on the 6RL^Ar^ arms were disappeared (Figures 3C,I). Line 21F3 contained two deleted translocation chromosomes T6BS.6RL^Ar^-2, carrying one Oligo-pSc200 signal band and two intercalary Oligo-pSc119.2-1 signal bands (Figures 3D,I). Line 21F7 contained a pair of deleted translocation chromosomes T6BS.6RL^Ar^-3, and the signals of Oligo-pSc200 on telomeric regions were disappeared (Figures 3E,I). In line 21F11, probe Oligo-pSc200 produced signals on the telomeric regions of the short arms of chromosomes 6A (Figures 3F–I). Therefore, line 21F11 contained a pair of small-segment translocations T6RL^Ar^-6AS.6AL and a pair of T6BS.6RL^Ar^-3 (Figures 3F–I). The weak GISH signals on the chromosomes T6RL^Ar^-6AS.6AL indicated that the segment of 6RL^Ar^ involved in translocation is small (Figures 3H,I).

### The Physical Location of 6RL^Ar^-Specific Markers

**Figure 4:**
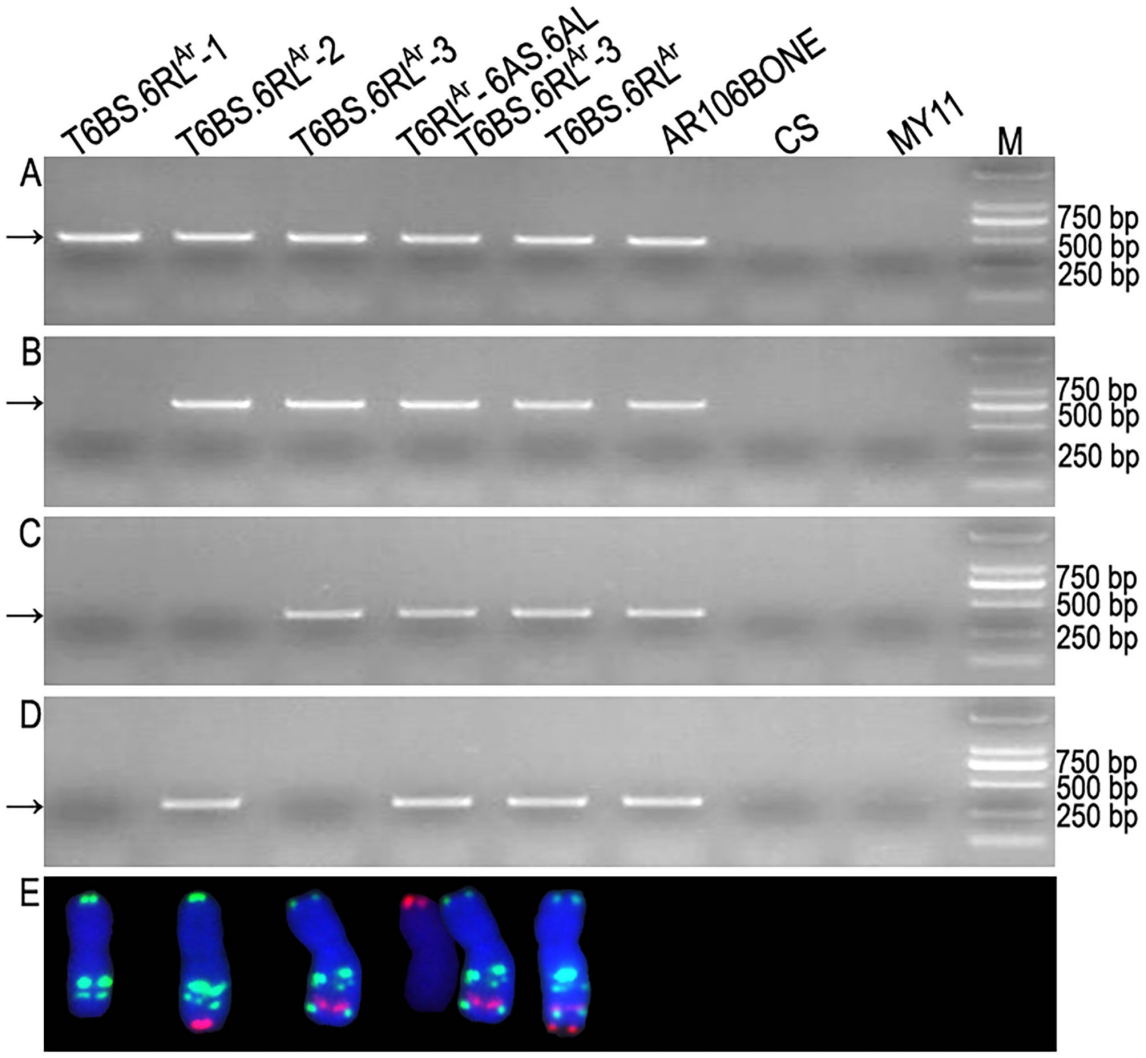
Physically mapped 6RL^Ar^-specific markers using deleted T6BS.6RL^Ar^ translocations. **(A)** Products amplified by primer pair Lo7.6RL-2 represent the markers located on T6BS.6RL^Ar^ and all the deleted T6BS.6RL^Ar^. **(B)** Products amplified by primer pair Lo7.6RL-74 represent the markers located on T6BS.6RL^Ar^, T6BS.6RL^Ar^-2, and T6BS.6RL^Ar^-3. **(C)** Products amplified by primer pair Ku.6RL-260 represent the markers located on T6BS.6RL^Ar^ and T6BS.6RL^Ar^-3. **(D)** Products amplified by primer pair Lo7.6RL-183 represent the markers located on T6BS.6RL^Ar^, T6BS.6RL^Ar^-2, and T6RL^Ar^-6AS.6AL. **(E)** Cut-and-paste translocation chromosomes corresponding to their amplified products in each electrophoresis lane. Arrows indicate the target bands amplified by 6RL^Ar^-specific markers. Common wheat Chinese Spring (CS), Mianyang11 (MY11), and rye AR106BONE were used as controls. M: DNA marker.

**Figure 5:**
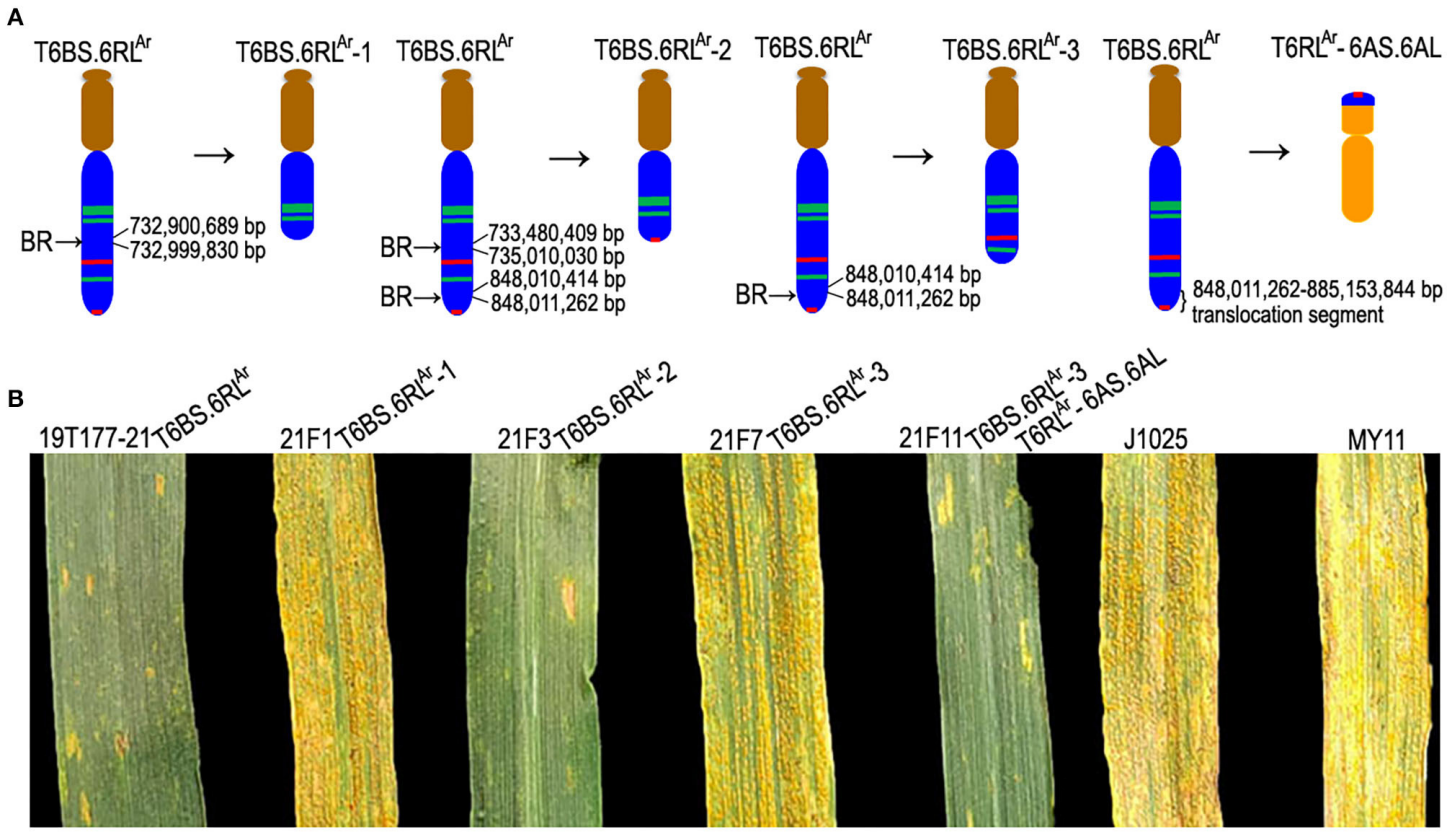
Ideograms of wheat-rye 6RL translocation chromosomes and stripe rust response test. **(A)** Ideograms of T6BS.6RL^Ar^, deleted T6BS.6RL^Ar^, and the small-segment translocation T6RL^Ar^-6AS.6AL. Brown, blue, and orange indicate segments of 6B, 6R, and 6A chromosomes, respectively. Green and red bands indicate the signals of Oligo-pSc119.2-1 and Oligo-pSc200, respectively. “BR” indicates a break point. The right brace indicates the 6RL^Ar^ segment that was transferred on the 6A chromosome. **(B)** Parental wheat J1025 and MY11 and lines 21F1 and 21F7 were highly susceptible to stripe rust and lines 19T177-21, 21F3, and 21F11 were highly resistant.

Among the 423 newly designed primer pairs, 204 amplified specific bands from T6BS.6RL^Ar^ translocation line and rye AR106BONE, but not from CS and MY11, indicated that these 204 primer pairs are 6RL^Ar^-specific markers (Figure 4 and Supplementary Table S1). Additionally, 95 of the 124 SLAF-seq markers amplified target bands from both T6BS.6RL^Ar^ and AR106BONE. A total of 299 (204 + 95) 6RL^Ar^-specific markers were obtained, and their target amplification regions on the 6RL arm are listed in Supplementary Table S1. The breakpoints on the 6RL^Ar^ arms of T6BS.6RL^Ar^-1, T6BS.6RL^Ar^-2, and T6BS.6RL^Ar^-3 were determined using the 299 markers. In total, 103 of the 299 markers amplified 6RL^Ar^-specific bands from translocation T6BS.6RL^Ar^-1 (Figure 4A), and these markers covered the region of 6RL from 333,302,151 to 732,900,689 bp (Supplementary Table S1). The first distal marker (Lo7.6RL-71) that did not amplify products from T6BS.6RL^Ar^-1 occupied the region from 732,999,830 to 733,000,692 bp (Supplementary Table S1). Therefore, the breakpoint in T6BS.6RL^Ar^-1 was located in the region of the 6RL^Ar^ arm between 732,900,689 and 732,999,830 bp (Figure 5A and Supplementary Table S1). Totally, 118 and 52 markers that were distributed into two regions of 6RL from 333,302,151 to 733,480,409 bp and from 848,011,262 to 885,003,466 bp, respectively amplified target bands from T6BS.6RL^Ar^-2, and 129 markers that distributed the region of 6RL from 735,010,030 to 848,010,414 bp did not produce amplicons from this translocation chromosome (Figures 4B–D and Supplementary Table S1). It can be determined that the two breakpoints in T6BS.6RL^Ar^-2 occurred in the two regions of 6RL^Ar^ arm between 733,480,409 and 735,010,030 bp and between 848,010,414 and 848,011,262 bp (Figure 5A and Supplementary Table S1). For T6BS.6RL^Ar^-3 translocation, 247 markers that were distributed on the 6RL region from 333,302,151 to 848,010,414 bp amplified target bands, and the rest 52 markers, which were the same as those amplified products from T6BS.6RL^Ar^-2, did not amplify amplicons (Figure 4D and Supplementary Table S1). According to the amplification of these markers, the breakpoint regions on 6RL^Ar^ in the three deleted T6BS.6RL^Ar^ translocations can be determined (Figure 5A and Supplementary Table S1). For the T6BS.6RL^Ar^-3 chromosome, the breakpoint between 848,010,414 and 848,011,262 bp was the same as the second breakpoint in the T6BS.6RL^Ar^-2 chromosome (Figure 5A and Supplementary Table S1). Therefore, the segments from 732,999,830 bp to telomere, from 735,010,030 to 848,010,414 bp, and from 848,011,262 bp to telomere of 6RL^Ar^ were deleted from T6BS.6RL^Ar^-1, T6BS.6RL^Ar^-2, and T6BS.6RL^Ar^-3, respectively (Figure 5A). The 6RL^Ar^ segment deleted from T6BS.6RL^Ar^-3 was transferred to T6RL^Ar^-6AS.6AL (Figures 4, 5A).

### Location of Stripe Rust Resistance Genes on Segment of 6RL^Ar^

Stripe rust response test indicated that translocation lines T6BS.6RL^Ar^, 21F3 (T6BS.6RL^Ar^-2), and 21F11 (T6BS.6RL^Ar^-3 and T6RL^Ar^-6AS.6AL) were highly resistant to stripe rust (IT = 1), and translocation lines 21F1 (T6BS.6RL^Ar^-1) and 21F7 (T6BS.6RL^Ar^-3) were highly susceptible (IT = 9; Figure 5B). According to the resistance of 21F3, 21F7, and 21F11, and the 6RL^Ar^ segments existed in T6BS.6RL^Ar^-2, T6BS.6RL^Ar^-3, and T6RL^Ar^-6AS.6AL, it was determined that the 6RL^Ar^ segment in T6RL^Ar^-6AS.6AL carried the stripe rust resistance gene. Therefore, it was determined that the 6RL^Ar^ region from 848,371,946 bp to the telomere (885,153,844 bp) carried the stripe rust resistance gene. Gene annotation indicated that there are 43 potential resistance genes located in the region from 848,011,885 to 885,153,844 bp of chromosome 6R (Supplementary Table S2). Only one of the 43 genes belongs to the kinase family protein gene and the other 42 genes are nucleotide-binding site and leucine-rich repeat (NBS-LRR)-like resistance protein genes (Supplementary Table S2).

### The Similarity of the Candidate Genes on 6RL Arms of Lo7 and AR106BONE

**Figure 6:**
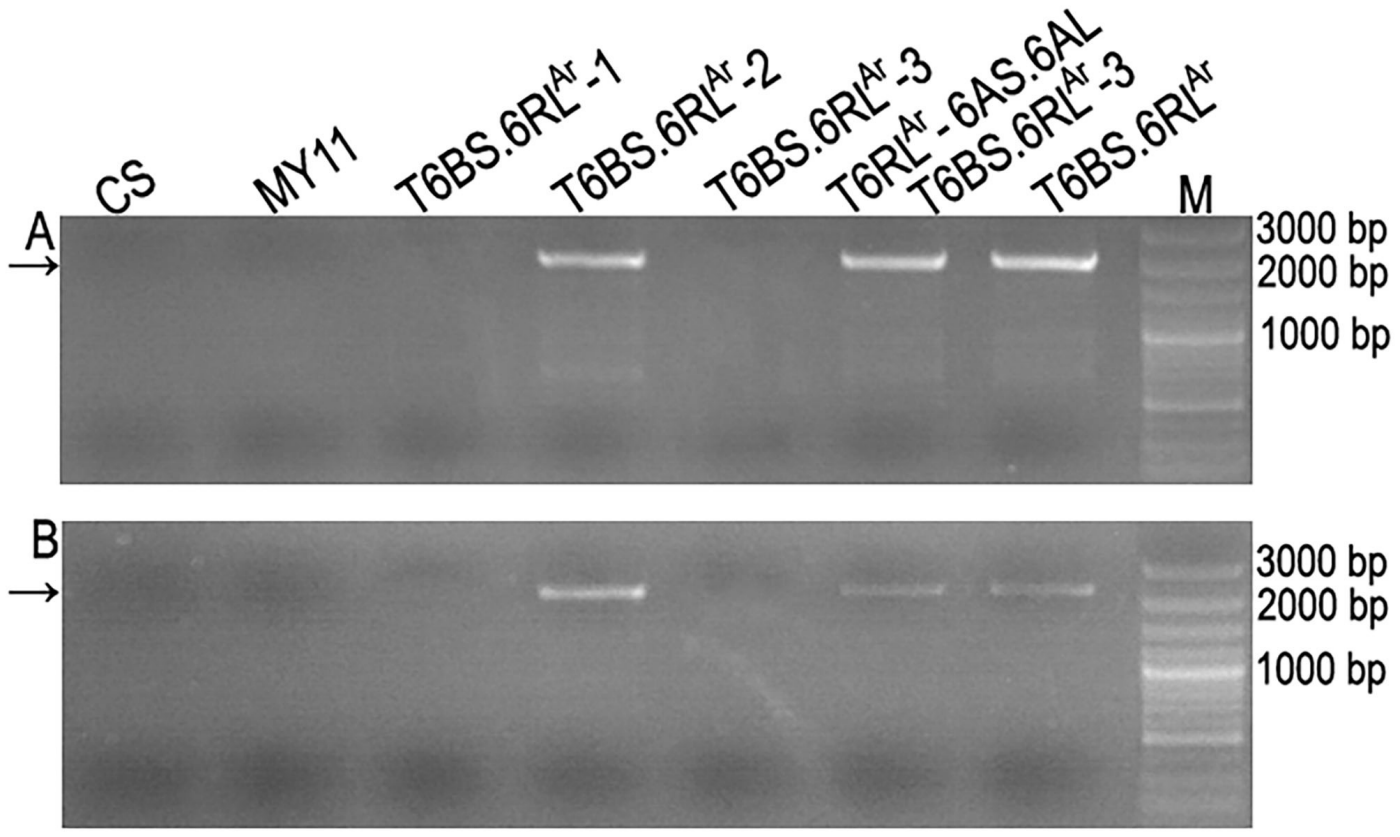
Amplification of partial segments of genes *SECCE6Rv1G0449960.1* and *SECCE6Rv1G0453070.1*. **(A)** Products amplified by primer pair P60. **(B)** Products amplified by primer pair P70. Arrows indicate the target bands amplified by the two primer pairs. Common wheat Chinese Spring (CS) and Mianyang11 (MY11) were used as controls. M: DNA marker.

The two primer pairs P60 and P70 amplified specific bands from translocation chromosomes T6BS.6RL^Ar^, T6BS.6RL^Ar^-2, and T6RL^Ar^-6AS.6AL but not from T6BS.6RL^Ar^-1 and T6BS.6RL^Ar^-3 translocations (Figure 6). The two primer pairs also did not amplify products from CS and MY11 (Figure 6). The sequences amplified from T6RL^Ar^-6AS.6AL were cloned and sequenced. The sequences amplified by primer pairs P60 and P70 were named Ar-60.1 (1,857 bp) and Ar-70.1 (2,423 bp), respectively (Supplementary Figures S1, S2). Sequences Ar-60.1 and Ar-70.1 had 99.03 and 99.50% similarity to the corresponding segments of genes *SECCE6Rv1G0449960.1* and *SECCE6Rv1G0453070.1*, respectively (Supplementary Figures S1, S2). These results indicated the conservation of resistance genes on the 6RL arms of Lo7 and AR106BONE.

## Discussion

### Polymorphism of 6R Chromosomes

The complex cytological structure of the 6R chromosome has been reported. It was known that the long arm of the rye 6R chromosome contains homologous groups 3 and 7 segments in the distal region (Devos et al., 1993; Li et al., 2013). FISH signal patterns of tandem repeats pSc119.2 on *S. cereale* 6R and *S. africanum* 6R^afr^ were different, and this indicated the structural alteration between the two kinds of 6R chromosomes (Li et al., 2020a). In this study, the cytological structure of 6R chromosomes derived from rye AR106BONE and Kustro is different. Some 6RL^Ku^-specific SLAF-seq markers that did not amplify products from 6R^Ar^ also exhibited the diversity between chromosomes 6R^Ku^ and 6R^Ar^. The 6RL arm with stripe rust resistance gene *Yr83* carried weak pSc119.2 signals in the intercalary and telomeric regions (Li et al., 2020b), and it is different from that of the 6RL^Ar^ arm. However, the pSc119.2 signal pattern of 6RL^Ar^ is similar to that on the 6RL arm of rye cv. Qinling (Hao et al., 2018). These results exhibited the abundant genetic diversity of chromosome 6R in the genus *Secale*. Therefore, it is necessary to continue to study the allelic variations of 6R chromosomes as they may enrich the genetic diversity of the resistance genes on them. Especially, the difference among the stripe rust resistance genes on different 6R chromosomes needs to be confirmed. Additionally, the results in this study make us think about whether the stripe rust resistance of the 6RL segment is controlled by a single gene or multiple genes.

### Using 6R Deletions to Locate Stripe Rust Resistance Genes

Rye chromosome deletion lines are useful for the location of resistance genes. The powdery mildew resistance gene *Pm56* was located in the subtelomeric region of the 6RS arm using 6R deletion lines (Hao et al., 2018). The stripe rust resistance gene *Yr83* was mapped to the bin of FL0.73-1.00 of 6RL using deletion mapping (Li et al., 2020b). Similarly, 6R chromosome deletion and translocation lines were used to locate the powdery mildew resistance gene on the 6RL arm at FL0.85-1.00 and the stripe rust resistance gene on the 6RS arm at FL0.95-1.00 (Li et al., 2020a). However, the detailed information about the physical location of these reported resistance genes on 6R chromosomes is still unclear. In this study, 6RL-specific markers have been anchored to the exact physical positions on the 6R chromosome using reference genomic sequences of rye, and these markers combined with deleted T6BS.6RL translocation chromosomes were used to reveal that the 6RL segment with stripe rust resistance gene(s) is about 37 Mb. The primer sequences of the 33 markers that were located in the bin with the *Yr83* gene were used for nucleotide BLAST searches against the 6R sequence of rye Lo7 (Rabanus-Wallace et al., 2021) using a BLAST tool in the Triticeae Multi-omics Center (http://202.194.139.32/), and these markers covered the 6R segment between 743,894,430 and 880,064,740 bp. Therefore, in this study, the 6RL segment with stripe rust resistance is smaller than that reported by Li et al. (2020b), and this narrows down the candidate genes with stripe rust resistance on 6R chromosome. The high similarity of the candidate resistance gene sequences between rye Lo7 and AR106BONE indicated that the physical mapping based on the reference genome of rye Lo7 can be applied to 6RL^Ar^ arm. Additionally, the new markers developed in this study enrich the 6R-specific molecular markers. It is a pity that the effect of the T6RL^Ar^-6AS.6AL chromosome on agronomic traits is unclear because it exists in a plant together with the T6BS.6RL^Ar^-3 chromosome. To get rid of the T6BS.6RL^Ar^-3, the cross between line 21F11 and some wheat lines that are susceptible to stripe rust has already been carried out. The T6RL^Ar^-6AS.6AL chromosome is a promising small-segment translocation for the improvement of wheat cultivars.

## Data Availability Statement

The original contributions presented in the study are included in the article/Supplementary Material, further inquiries can be directed to the corresponding author/s.

## Author Contributions

ZT and SF conceived and designed the study, created the materials, analyzed the data, and wrote the manuscript. YD and JL performed the experiments and analyzed the data. ZY and GL created the materials. All authors read and approved the final manuscript.

## Funding

This manuscript was provided by the National Natural Science Foundation of China (no. 31770373), the Open Project Program of the State Key Laboratory of Plant Cell and Chromosome Engineering, Institute of Genetics and Developmental Biology, China (no. PCCE-KF-2021-02), and the Sichuan Science and Technology Program (no. 2022ZDZX0014).

## Conflict of Interest

The authors declare that the research was conducted in the absence of any commercial or financial relationships that could be construed as a potential conflict of interest.

## Publisher's Note

All claims expressed in this article are solely those of the authors and do not necessarily represent those of their affiliated organizations, or those of the publisher, the editors and the reviewers. Any product that may be evaluated in this article, or claim that may be made by its manufacturer, is not guaranteed or endorsed by the publisher.

## Supplementary Material

Supplementary Figure S1 Alignment between the sequence Ar-60.1 and Lo7-60.1. Ar-60.1 represents the sequence amplified by P60 from T6RL^Ar^-6AS.6AL translocation. Lo7-60.1 represents the partial sequence of *SECCE6Rv1G0449960.1* of rye Lo7. Red arrows indicate the primer sequences.

Supplementary Figure S2 Alignment between the sequence Ar-70.1 and Lo7-70.1. Ar-70.1 represents the sequence amplified by P70 from T6RL^Ar^-6AS.6AL translocation. Lo7-70.1 represents the partial sequence of *SECCE6Rv1G0453070.1* of rye Lo7. Red arrows indicate the primer sequences.
