# Supplementary figures and images for: The Physical Location of Stripe Rust Resistance Genes on Chromosome 6 of Rye (Secale cereale L.) AR106BONE
Source: Front Plant Sci. 2022 Jun 29;13:928014. doi: 10.3389/fpls.2022.928014 (PMC9277549; doi:10.3389/fpls.2022.928014)

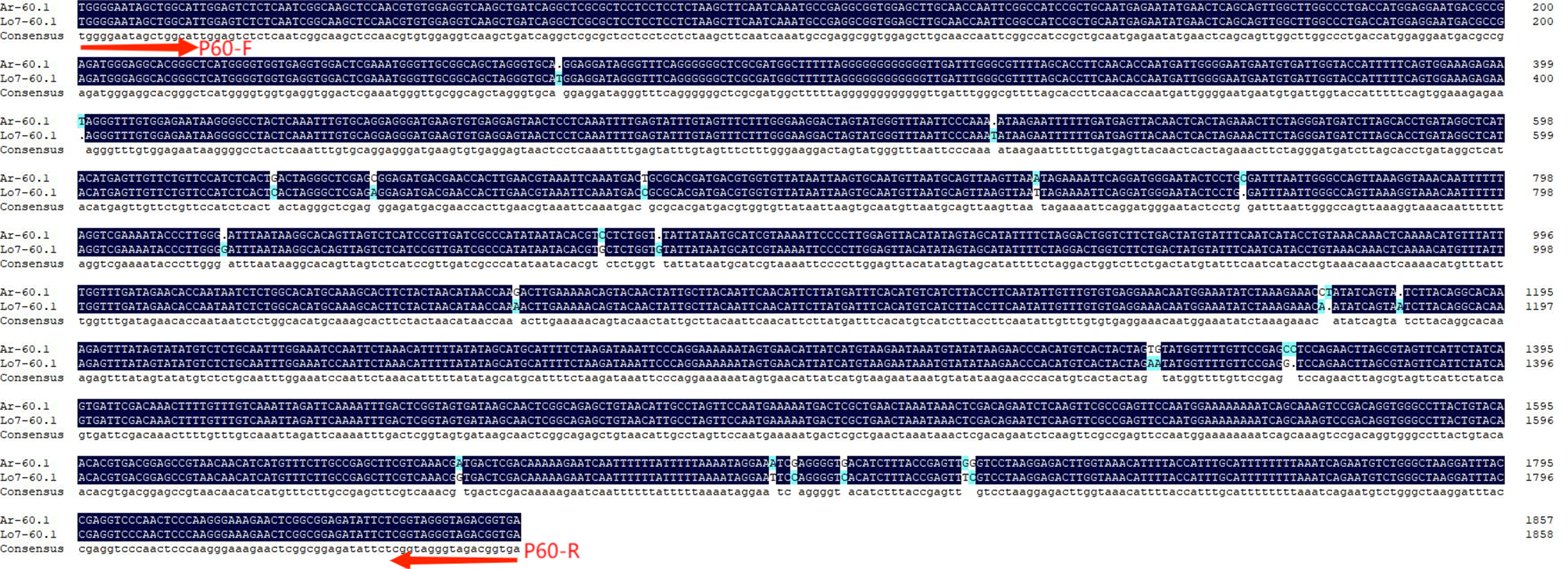

Supplement: Supplementary Figure S1 — Alignment between the sequence Ar-60.1 and Lo7-60.1. Ar-60.1 represents the sequence amplified by P60 from T6RLAr-6AS.6AL translocation. Lo7-60.1 represents the partial sequence of SECCE6Rv1G0449960.1 of rye Lo7. Red arrows indicate the primer sequences. [file Image_1.TIF]

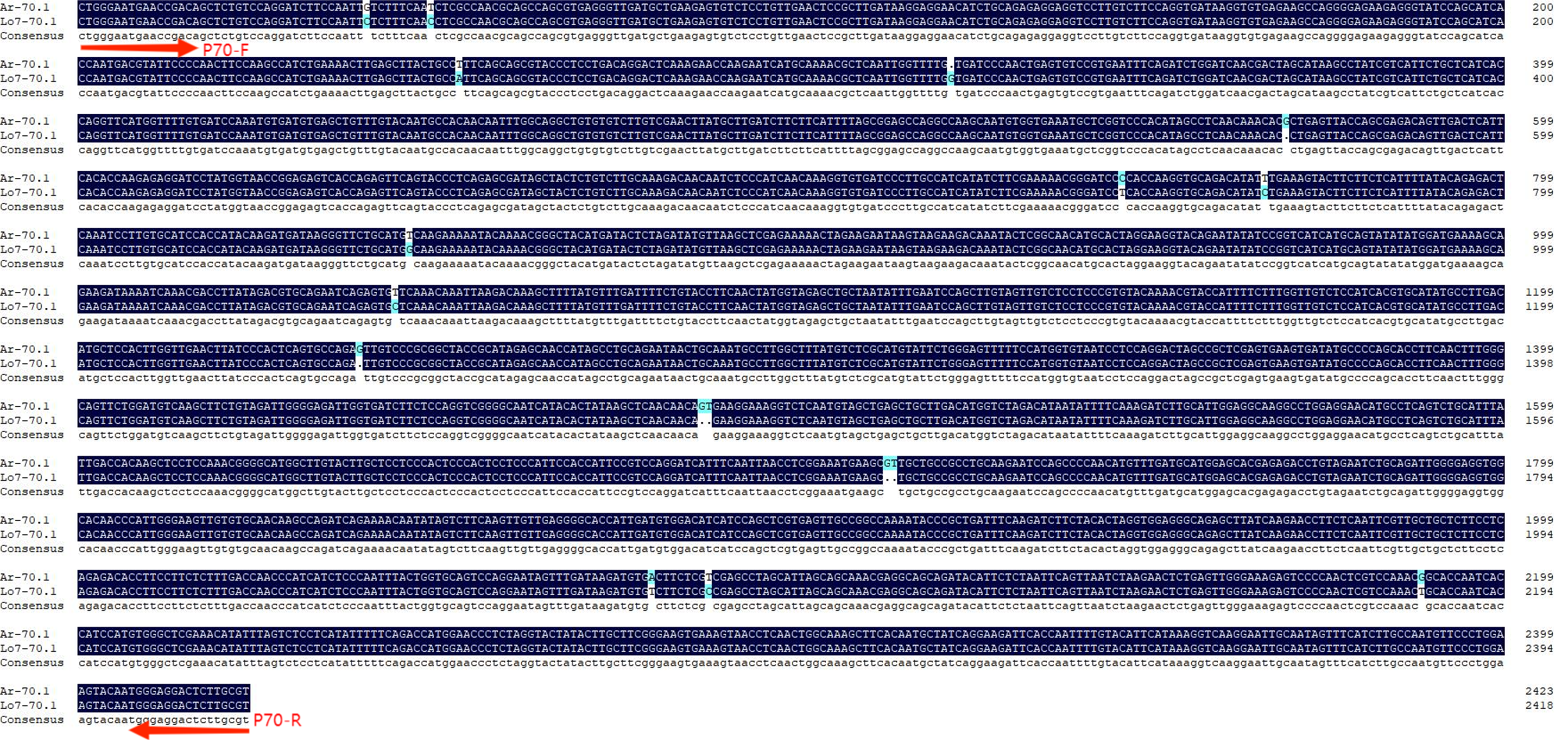

Supplement: Supplementary Figure S2 — Alignment between the sequence Ar-70.1 and Lo7-70.1. Ar-70.1 represents the sequence amplified by P70 from T6RLAr-6AS.6AL translocation. Lo7-70.1 represents the partial sequence of SECCE6Rv1G0453070.1 of rye Lo7. Red arrows indicate the primer sequences. [file Image_2.TIF]
