# Supplementary material for: The Physical Location of Stripe Rust Resistance Genes on Chromosome 6 of Rye (Secale cereale L.) AR106BONE
Source: Front Plant Sci. 2022 Jun 29;13:928014. doi: 10.3389/fpls.2022.928014 (PMC9277549; doi:10.3389/fpls.2022.928014)
Supplement: Supplementary file 4 [file Table_2.DOCX]

Supplementary Table S2. Potential disease resistance genes in 6RL region with resistance to stripe rust

| Gene | Predicted protein | Position on 6R (bp) | Gene size (bp) |
| --- | --- | --- | --- |
| *SECCE6Rv1G0446870.1* | NBS-LRR-like resistance protein | chr6R:846,535,519 to 846,555,318 | 19,800 |
| *SECCE6Rv1G0447170.1* | NBS-LRR-like resistance protein | chr6R:849,208,861 to 849,210,488 | 1,628 |
| *SECCE6Rv1G0447250.1* | NBS-LRR-like resistance protein | chr6R:849,688,006 to 849,689,546 | 1,541 |
| *SECCE6Rv1G0447260.1* | NBS-LRR-like resistance protein | chr6R:849,689,881 to 849,690,832 | 952 |
| *SECCE6Rv1G0447640.1* | NBS-LRR-like resistance protein | chr6R:851,206,291 to 851,208,053 | 1,763 |
| *SECCE6Rv1G0447660.1* | NBS-LRR-like resistance protein | chr6R:851,226,094 to 851,229,858 | 3,765 |
| *SECCE6Rv1G0447710.1* | NBS-LRR-like resistance protein | chr6R:851,253,911 to 851,255,179 | 1,269 |
| *SECCE6Rv1G0447810.1* | NBS-LRR-like resistance protein | chr6R:851,762,334 to 851,768,000 | 5,667 |
| *SECCE6Rv1G0447820.1* | NBS-LRR-like resistance protein | chr6R:851,842,942 to 851,843,684 | 743 |
| *SECCE6Rv1G0447860.1* | NBS-LRR-like resistance protein | chr6R:852,184,717 to 852,190,130 | 5,414 |
| *SECCE6Rv1G0447930.1* | NBS-LRR-like resistance protein | chr6R:852,448,893 to 852,452,510 | 3,618 |
| *SECCE6Rv1G0447940.1* | NBS-LRR-like resistance protein | chr6R:852,577,143 to 852,580,769 | 3,627 |
| *SECCE6Rv1G0448200.1* | NBS-LRR-like resistance protein | chr6R:854,589,350 to 854,589,813 | 464 |
| *SECCE6Rv1G0449370.1* | NBS-LRR-like resistance protein | chr6R:861,699,432 to 861,702,411 | 2,980 |
| *SECCE6Rv1G0449380.1* | NBS-LRR-like resistance protein | chr6R:861,702,424 to 861,704,561 | 2,138 |
| *SECCE6Rv1G0449770.1* | NBS-LRR-like resistance protein | chr6R:864,011,665 to 864,030,395 | 18,731 |
| *SECCE6Rv1G0449960.1* | NBS-LRR-like resistance protein | chr6R:865,172,763 to 865,182,235 | 9,473 |
| *SECCE6Rv1G0449920.1* | NBS-LRR-like resistance protein | chr6R:865,140,383 to 865,142,953 | 2,571 |
| *SECCE6Rv1G0450040.1* | NBS-LRR-like resistance protein | chr6R:865,626,675 to 865,630,526 | 3,852 |
| *SECCE6Rv1G0450040.1* | NBS-LRR-like resistance protein | chr6R:865,626,675 to 865,630,526 | 3,852 |
| *SECCE6Rv1G0450160.1* | NBS-LRR-like resistance protein | chr6R:866,721,133 to 866,727,277 | 6,145 |
| *SECCE6Rv1G0450200.1* | Kinase family protein | chr6R:866,913,695 to 866,914,748 | 1,054 |
| SECCE6Rv1G0450920.1 | NBS-LRR-like resistance protein | chr6R:870,376,781 to 870,379,943 | 3,163 |
| SECCE6Rv1G0450930.1 | NBS-LRR-like resistance protein | chr6R:870,446,950 to 870,450,112 | 3,163 |
| SECCE6Rv1G0450950.1 | NBS-LRR-like resistance protein | chr6R:870,523,022 to 870,524,505 | 1,484 |
| SECCE6Rv1G0451020.1 | NBS-LRR-like resistance protein | chr6R:870,756,612 to 870,760,299 | 3,688 |
| SECCE6Rv1G0451080.1 | NBS-LRR-like resistance protein | chr6R:871,237,984 to 871,238,460 | 477 |
| SECCE6Rv1G0451090.1 | NBS-LRR-like resistance protein | chr6R:871,243,998 to 871,247,366 | 3,369 |
| SECCE6Rv1G0451100.1 | NBS-LRR-like resistance protein | chr6R:871,254,160 to 871,258,049 | 3,890 |
| SECCE6Rv1G0451110.1 | NBS-LRR-like resistance protein | chr6R:871,329,382 to 871,333,627 | 4,246 |
| SECCE6Rv1G0452840.1 | NBS-LRR-like resistance protein | chr6R:879,491,681 to 879,494,376 | 2,696 |
| SECCE6Rv1G0452810.1 | NBS-LRR-like resistance protein | chr6R:879,459,960 to 879,463,700 | 3,741 |
| SECCE6Rv1G0452850.1 | NBS-LRR-like resistance protein | chr6R:879,494,491 to 879,496,485 | 1,995 |
| SECCE6Rv1G0452890.1 | NBS-LRR-like resistance protein | chr6R:879,616,217 to 879,618,674 | 2,458 |
| SECCE6Rv1G0452970.1 | NBS-LRR-like resistance protein | chr6R:879,719,594 to 879,720,079 | 486 |
| SECCE6Rv1G0452900.1 | NBS-LRR-like resistance protein | chr6R:879,618,684 to 879,620,958 | 2,275 |
| SECCE6Rv1G0452960.1 | NBS-LRR-like resistance protein | chr6R:879,717,090 to 879,719,515 | 2,426 |
| SECCE6Rv1G0452940.1 | NBS-LRR-like resistance protein | chr6R:879,714,326 to 879,715,244 | 919 |
| SECCE6Rv1G0452950.1 | NBS-LRR-like resistance protein | chr6R:879,715,462 to 879,717,076 | 1,615 |
| SECCE6Rv1G0453000.1 | NBS-LRR-like resistance protein | chr6R:879,800,294 to 879,804,006 | 3,713 |
| SECCE6Rv1G0453030.1 | NBS-LRR-like resistance protein | chr6R:879,869,819 to 879,878,231 | 8,413 |
| SECCE6Rv1G0453070.1 | NBS-LRR-like resistance protein | chr6R:880,055,830 to 880,061,327 | 5,498 |
| SECCE6Rv1G0453720.1 | NBS-LRR-like resistance protein | chr6R:884,447,063 to 884,448,862 | 1,800 |
| SECCE6Rv1G0453660.1 | NBS-LRR-like resistance protein | chr6R:884,217,108 to 884,218,922 | 1,815 |
